# SRRM4-dependent neuron-specific alternative splicing of protrudin transcripts regulates neurite outgrowth

**DOI:** 10.1038/srep41130

**Published:** 2017-01-20

**Authors:** Takafumi Ohnishi, Michiko Shirane, Keiichi I. Nakayama

**Affiliations:** 1 Department of Molecular and Cellular Biology, Medical Institute of Bioregulation, Kyushu University, 3-1-1 Maidashi, Higashi-ku, Fukuoka, Fukuoka 812-8582, Japan

## Abstract

Alternative splicing gives rise to diversity of the proteome, and it is especially prevalent in the mammalian nervous system. Indeed, many factors that control the splicing process govern nervous system development. Among such factors, SRRM4 is an important regulator of aspects of neural differentiation including neurite outgrowth. The mechanism by which SRRM4 regulates neurite outgrowth has remained poorly understood, however. We now show that SRRM4 regulates the splicing of protrudin gene (*Zfyve27*) transcripts in neuronal cells. SRRM4 was found to promote splicing of protrudin pre-mRNA so as to include a microexon (exon L) encoding seven amino acids in a neuron-specific manner. The resulting protein (protrudin-L) promotes neurite outgrowth during neurogenesis. Depletion of SRRM4 in Neuro2A cells impaired inclusion of exon L in protrudin mRNA, resulting in the generation of a shorter protein isoform (protrudin-S) that is less effective at promoting neurite extension. SRRM4 was found to recognize a UGC motif that is located immediately upstream of exon L and is necessary for inclusion of exon L in the mature transcript. Deletion of exon L in Neuro2A or embryonic stem cells inhibited neurite outgrowth. Our results suggest that SRRM4 controls neurite outgrowth through regulation of alternative splicing of protrudin transcripts.

Alternative splicing is a process by which different pairs of splice sites are selected to generate multiple transcripts from a single gene^1^,^2^,^3^. It has contributed greatly to expansion of structural and functional complexity of the proteome through evolution, and it is especially prevalent in the vertebrate nervous system, playing key roles in neurogenesis, synaptogenesis, neurite outgrowth, axon guidance, ion channel activity, and long-term potentiation^4^,^5^,^6^. The interplay between trans-acting factors and corresponding cis-acting elements in transcripts allows precise control of tissue- and developmental stage–specific splicing events^4^,^7^,^8^.

During neurogenesis, neurons are generated from neural stem or progenitor cells (NPCs). This process is characterized by changes in transcript and protein abundance as well as by those in cell morphology^9^. Neural-specific splicing regulators—including Nova, Rbfox, Ptbp, SR proteins, and SR-related proteins—play important roles in neurogenesis and in maintenance of neuronal activities^10^,^11^,^12^,^13^,^14^,^15^,^16^,^17^,^18^,^19^. The SR protein family comprises several phylogenetically conserved and structurally related proteins with a characteristic domain rich in arginine and serine residues known as the RS domain^18^. Members of this protein family either repress or promote the formation of active splicing complexes, often depending on the location of cognate binding sites within exon or intron sequences^18^. The SR-related proteins also contain RS domains but may or may not possess RNA binding domains^19^. Phosphorylation of RS domains is thought to be fundamental to protein-protein and protein-RNA interactions required for spliceosome assembly. About 40 mammalian RS domain–containing proteins contribute to the splicing reaction^20^. SRRM4 is an SR-related protein that is specifically expressed in nervous tissue and cells^17^,^21^,^22^,^23^,^24^. It was shown to promote the inclusion of 30% to 50% of the conserved cassette alternative exons that manifest a brain-specific inclusion pattern in mouse^24^. Depletion of SRRM4 in Neuro2A cells resulted in impairment of neurite outgrowth, whereas its ablation in mouse embryos prevented differentiation of neuronal progenitors in the cortex^17^,^21^. These observations suggest that SRRM4 is a key factor in neural differentiation. The mechanism by which loss of SRRM4 in neurons impairs neurite outgrowth and the consequent development of neuronal polarity has remained unclear, however.

Protrudin (encoded by the *Zfyve27* gene) is a membrane protein that regulates polarized vesicular transport^25^,^26^,^27^. Protrudin-L is a neuron-specific isoform that contains an additional seven amino acids compared with the short isoform (protrudin-S) as a result of alternative splicing^28^. These seven amino acids are encoded by a microexon (exon L) whose inclusion in transcripts is restricted to neural tissue^28^. Protrudin-L promotes neurite outgrowth and the development of neuronal polarity more efficiently than does protrudin-S. Given that the additional seven amino acids encoded by exon L in protrudin-L are located adjacent to an FFAT (two phenylalanines in an acidic tract) motif that is essential for the interaction of protrudin-L with vesicle-associated membrane protein–associated protein (VAP), inclusion of these residues likely affects the binding of protrudin to VAP as well as the polarized vesicle transport that supports neurite extension^26^,^28^. The mechanism by which the inclusion of exon L in protrudin transcripts is restricted to the nervous system has remained unknown.

Depletion of SRRM4 in Neuro2A cells was shown to down-regulate the abundance of protrudin-L mRNA, and mice deficient in SRRM4 function as a result of deletion of exons 7 and 8 of the SRRM4 gene manifest a reduced extent of exon L inclusion^17^. These observations suggest that SRRM4 regulates the inclusion of exon L in protrudin transcripts. It has been unclear, however, whether SRRM4 directly promotes the inclusion of exon L. The extent to which protrudin-L generation contributes to the process of SRRM4-dependent neurite outgrowth during neurogenesis has also been unknown.

We now show that SRRM4 recognizes a UGC motif flanking exon L of protrudin pre-mRNA and thereby promotes the inclusion of this exon in the mature mRNA in neural cells. Depletion of SRRM4 in Neuro2A cells resulted in impairment of neurite outgrowth that was associated with attenuation of protrudin-L generation and rescued by forced expression of protrudin-L. Furthermore, neurons differentiated from embryonic stem cells (ESCs) lacking exon L manifested a reduced axon/dendrite ratio. Together, our results thus reveal that neurite outgrowth is regulated by SRRM4-dependent alternative splicing of protrudin mRNA.

## Results

### Regulation of protrudin transcript splicing via a TGC motif located upstream of exon L

The neuron-specific long isoform of protrudin (protrudin-L) is produced by alternative splicing of transcripts to include the RNA region corresponding to a microexon (exon L) that is composed of 21 nucleotides and located between exons VIII and IX. Protrudin-L thus contains an additional seven amino acids compared with the ubiquitously expressed short isoform (protrudin-S). We previously showed that an atypical splice acceptor sequence flanking exon L is necessary for repression of inclusion of the neuron-specific exon L in nonneural tissues^28^. However, the cis-acting elements and corresponding trans-acting factors that promote the inclusion of exon L in neural cells have remained to be identified.

**Figure 1:**
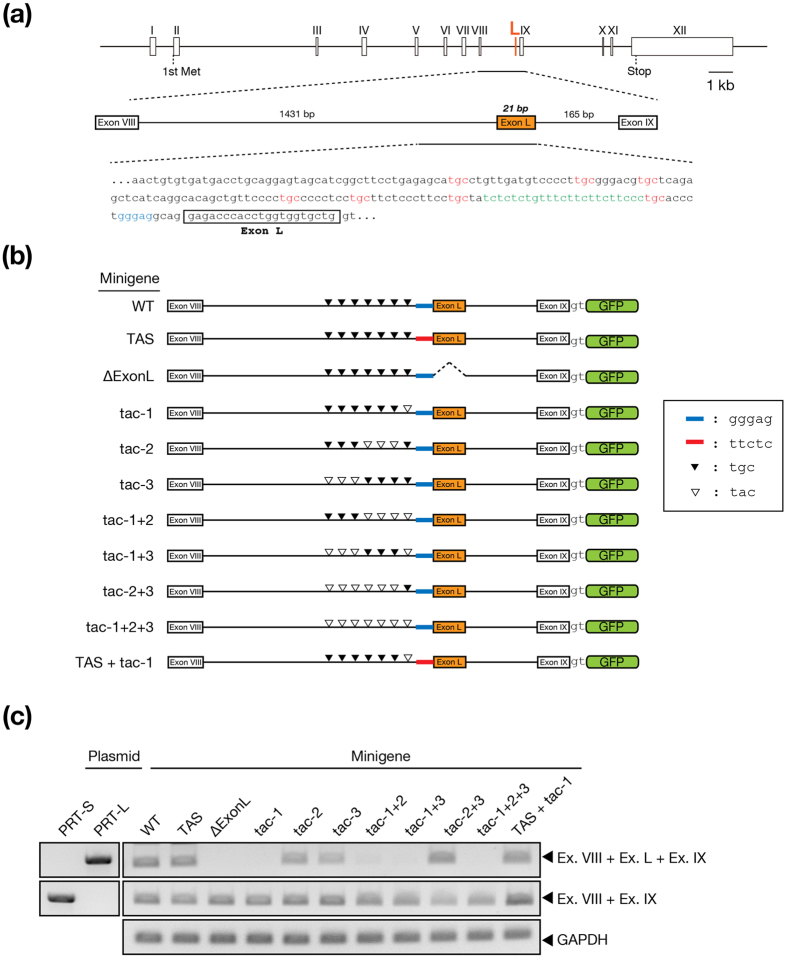
A TGC motif located upstream of exon L is necessary for inclusion of this exon in protrudin mRNA. (**a**) Structure of the mouse protrudin gene. Boxes indicate exons, with exon L, which encodes the additional sequence in protrudin-L, being shown in orange. The nucleotide sequence of exon L and its flanking region is also shown. Nucleotides shown in red constitute TGC motifs in the flanking region of exon L, those in blue form an atypical purine tract in the 3′ splice site of this exon, and those in green form a polypyrimidine tract. The boxed region in the nucleotide sequence indicates exon L. (**b**) Construction of splicing minigenes for protrudin. A genomic fragment spanning exons VIII and IX of the mouse protrudin gene was linked to cDNA encoding GFP with insertion of two nucleotides as indicated. Blue and red bars indicate the atypical sequence of the 3′ splice site of exon L and the corresponding typical sequence, respectively. Filled and unfilled arrowheads indicate the native TGC and mutant TAC sequences, respectively. (**c**) RT-PCR analysis of total RNA from Neuro2A cells infected with retroviruses containing the splicing minigenes for protrudin. PCR was performed with primer pairs targeted to protrudin-S (Ex. VIII + Ex. IX), protrudin-L (Ex. VIII + Ex. L + Ex. IX), and glyceraldehyde-3-phosphate dehydrogenase (GAPDH, control) cDNAs. Lanes PRT-S and PRT-L contain PCR products derived from plasmid DNA corresponding to protrudin-S or protrudin-L, respectievly, as controls.

SRRM4 is an SR-related protein that promotes inclusion of neural-specific exons in target mRNAs^21^. It recognizes a UGC motif located between a polypyrimidine tract and the targeted exon in the pre-mRNA and thereby stabilizes the spliceosome^22^. The intron region upstream of exon L contains seven TGC motifs that might be expected to give rise to SRRM4 binding sites (Fig. 1a). To investigate whether these TGC motifs are responsible for the selection of exon L in neural cells, we constructed a minigene containing a 1.8-kb genomic fragment of the mouse protrudin gene that spans the 3′ region of exon VIII to the 5′ region of exon IX and which is fused to the coding sequence for green fluorescent protein (GFP) (Fig. 1b). Wild-type (WT) or mutant versions of the minigene were introduced into Neuro2A cells by retroviral infection, and the spliced transcripts derived from each minigene were examined by reverse transcription (RT) and polymerase chain reaction (PCR) analysis (Fig. 1c). Production of the long transcript containing the exon L sequence was detected in cells expressing the WT minigene or a mutant minigene in which the atypical acceptor sequence was replaced with a typical acceptor sequence (TAS), but not in those expressing a mutant minigene in which exon L was deleted (∆ExonL) or in which all seven TGC motifs were replaced with TAC (tac-1 + 2 + 3). Further analysis revealed that minigenes in which the 3′-most TGC motif was replaced with TAC (tac-1, tac-1 + 2, and tac-1 + 3) also failed to generate the long transcript. In contrast, minigenes in which another three or the other six TGC motifs were replaced with TAC (tac-2, tac-3, and tac-2 + 3) maintained the ability to generate the long transcript. A mutant minigene in which the atypical acceptor sequence was replaced with a typical acceptor sequence and the 3′-most TGC motif was replaced with TAC (TAS + tac-1) also maintained the ability to generate the long transcript, suggesting that inclusion of exon L is controlled by both the atypical acceptor sequence and the 3′-most TGC motif. Overall, these results thus indicated that the presence of the 3′-most TGC motif is necessary and sufficient for inclusion of the region corresponding to exon L by alternative splicing.

### SRRM4 recognizes the essential UGC motif of protrudin pre-mRNA to promote exon L inclusion

**Figure 2:**
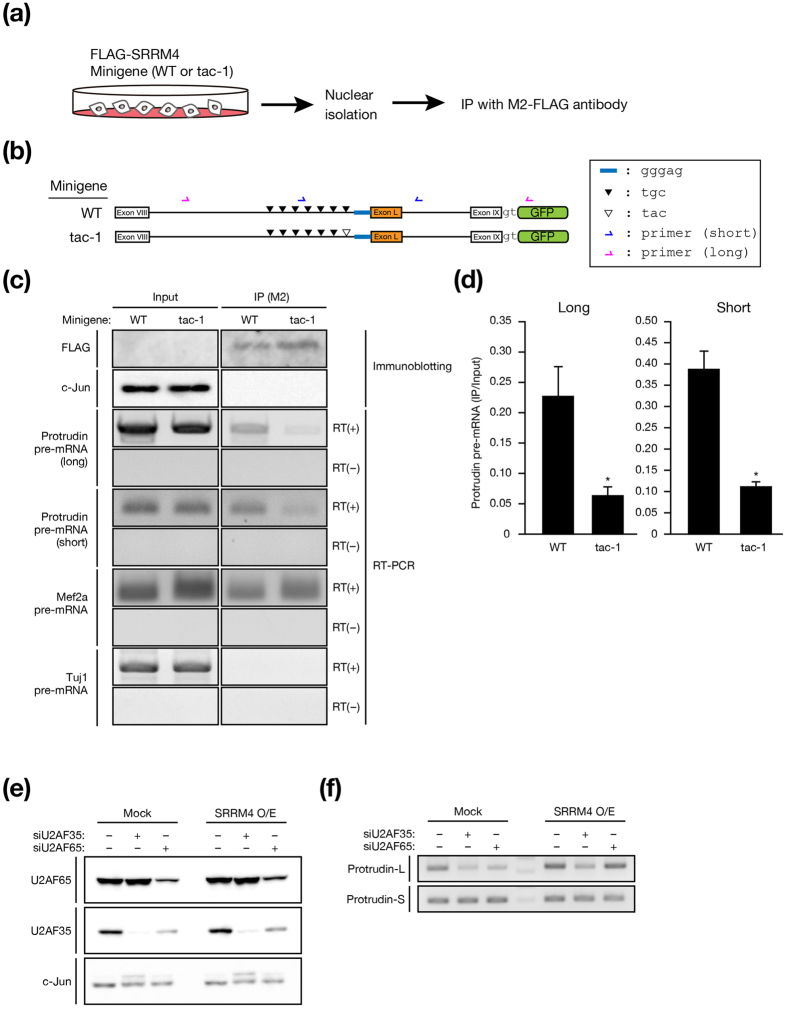
SRRM4 recognizes the UGC motif located immediately upstream of exon L in protrudin pre-mRNA. (**a**) Experimental protocol for RNA immunoprecipitation assays. Neuro2A cells stably expressing FLAG-SRRM4 and either the WT or tac-1 protrudin minigene were harvested for preparation of nuclear extracts, which were then subjected to immunoprecipitation (IP) with antibodies to FLAG (M2). (**b**) Primer sets for detection of protrudin pre-mRNA. Minigene constructs are presented as in Fig. 1b. Blue and red half-arrows indicate the primer recognition sites in the constructs. (**c**) Immunoprecipitated proteins and RNAs were subjected to immunoblot analysis with antibodies to FLAG and to c-Jun (negative control) as well as to RT-PCR analysis with primers specific for protrudin, Mef2a (positive control), or Tuj1 (negative control) pre-mRNAs. PCR was also performed without (–) prior RT as a control. (**d**) The intensities of the RT-PCR bands for protrudin pre-mRNA in experiments similar to that shown in **c** were analyzed with ImageJ software. Data are means ± s.e.m. from three independent experiments. *P < 0.05 (Student’s *t* test) versus corresponding value for WT. (**e**,**f**) Neuro2A cells transfected with control, U2AF35, or U2AF65 siRNAs as well as infected with a retrovirus encoding SRRM4 (O/E) or with the corresponding empty virus (Mock) were subjected to immunoblot (IB) analysis with antibodies to U2AF65, to U2AF35, and to c-Jun (loading control) (**e**) as well as to RT-PCR analysis with primers specific for protrudin-L and protrudin-S (**f**).

To examine whether SRRM4 indeed binds to the 3′-most UGC motif in the intron upstream of exon L of protrudin pre-mRNA, we performed RNA immunoprecipitation with nuclear extracts of Neuro2A cells expressing FLAG epitope–tagged SRRM4 and either the WT or tac-1 protrudin minigene (Fig. 2a). Immunoprecipitates prepared with antibodies to FLAG (M2) were then subjected to immunoblot analysis as well as to RT-PCR analysis with two sets of primers to detect long and short protrudin pre-mRNA fragments (Fig. 2b,c). Quantification of protrudin pre-mRNA showed that the amount of pre-mRNA bound to SRRM4 was reduced by mutation of the 3′-most TGC motif (Fig. 2d), suggesting that SRRM4 may interact directly or indirectly with this motif of protrudin pre-mRNA.

SRRM4 interacts with both U2AF35 and U2AF65 (ref. 22). We therefore examined whether the production of protrudin-L was affected by depletion of either of these splicing factors with specific small interfering RNAs (siRNAs) in Neuro2A cells (Fig. 2e). We found that the abundance of U2AF35 was also moderately reduced on depletion of U2AF65, suggesting that the stability of U2AF35 might be dependent on the presence of U2AF65. Production of protrudin-L protein was attenuated by depletion of either U2AF35 or U2AF65 in Neuro2A cells (Fig. 2f). Overexpression of SRRM4 prevented this effect of U2AF65 depletion but not that of U2AF35 depletion. Collectively, these results suggested that, although both U2AF35 and U2AF65 are required for the production of protrudin-L, SRRM4 may promote the corresponding splicing reaction by facilitating recognition of the polypyrimidine tract located upstream of exon L by U2AF65.

**Figure 3:**
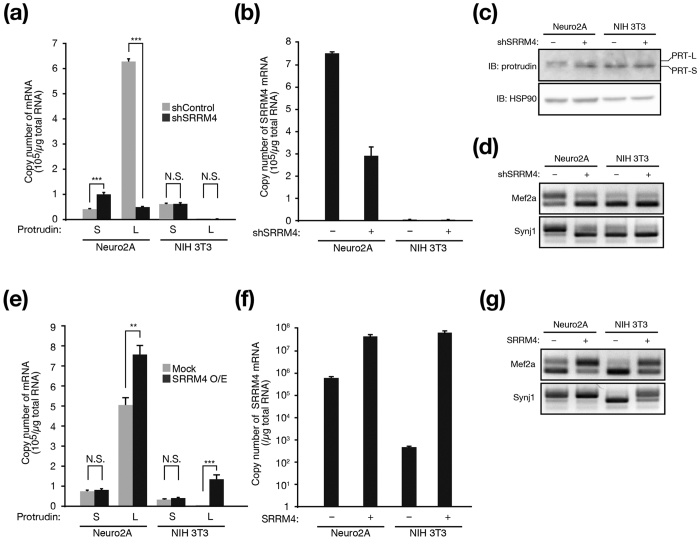
SRRM4 promotes the inclusion of exon L in protrudin mRNA. (**a**,**b**) Quantitative RT-PCR analysis of the absolute amounts of protrudin-S and protrudin-L mRNAs (**a**) as well as of SRRM4 mRNA (**b**) in Neuro2A and NIH 3T3 cells infected with retroviruses encoding control (shControl) or SRRM4 (shSRRM4) shRNAs. (**c**) Neuro2A and NIH 3T3 cells infected with retroviruses encoding shControl or shSRRM4 were subjected to immunoblot (IB) analysis with antibodies to protrudin and to HSP90 (loading control). The positions of bands corresponding to protrudin-L (PRT-L) and protrudin-S (PRT-S) are indicated. (**d**) RT-PCR analysis of Mef2a and Synj1 mRNAs in Neuro2A and NIH 3T3 cells infected with retroviruses as in **a** and **b**. (**e**,**f**) Quantitative RT-PCR analysis of the absolute amounts of protrudin-S and protrudin-L mRNAs (**e**) as well as of SRRM4 mRNA (**f**) in Neuro2A and NIH 3T3 cells infected with a retrovirus encoding SRRM4 (O/E) or with the corresponding empty virus (Mock). (**g**) RT-PCR analysis of Mef2a and Synj1 mRNAs in Neuro2A and NIH 3T3 cells infected with retroviruses as in (**e** and **f)**. All quantitative data are means ± s.e.m. for three independent experiments. **P < 0.01, ***P < 0.001 (Student’s *t* test). N.S., not significant.

To test the effect of SRRM4 on alternative splicing of protrudin transcripts, we depleted or overexpressed SRRM4 in Neuro2A cells (which predominantly express protrudin-L) and NIH 3T3 cells (which exclusively express protrudin-S) (Fig. 3a). The abundance of SRRM4 mRNA in Neuro2A cells was about 1000 times that in NIH 3T3 cells and was reduced by retrovirus-mediated introduction of a short hairpin RNA (shRNA) specific for SRRM4 mRNA (Fig. 3b). Immunoblot analysis showed that the abundance of protrudin-L was reduced whereas that of protrudin-S was increased by depletion of SRRM4 in Neuro2A cells (Fig. 3c). The exclusive expression of protrudin-S in NIH 3T3 cells was not affected by introduction of SRRM4 shRNA. As expected, exon inclusion for known targets of SRRM4 (Mef2a and Synj1 transcripts)^17^,^22^ was attenuated in Neuro2A cells depleted of SRRM4 (Fig. 3d). Knockdown of SRRM4 also significantly reduced the abundance of protrudin-L mRNA and increased that of protrudin-S mRNA in Neuro2A cells (Fig. 3a). Overexpression of SRRM4 (Fig. 3f) increased exon inclusion for Mef2a and Synj1 transcripts in both Neuro2A and NIH 3T3 cells (Fig. 3g). The abundance of protrudin-L mRNA was significantly increased, whereas that of protrudin-S mRNA was unaffected, by SRRM4 overexpression in both Neuro2A and NIH 3T3 cells (Fig. 3e). These results thus suggested that SRRM4 recognizes the UGC motif adjacent to exon L and may thereby promote exon L inclusion in protrudin transcripts in Neuro2A cells.

### SRRM4 regulates protrudin-dependent neurite outgrowth

**Figure 4:**
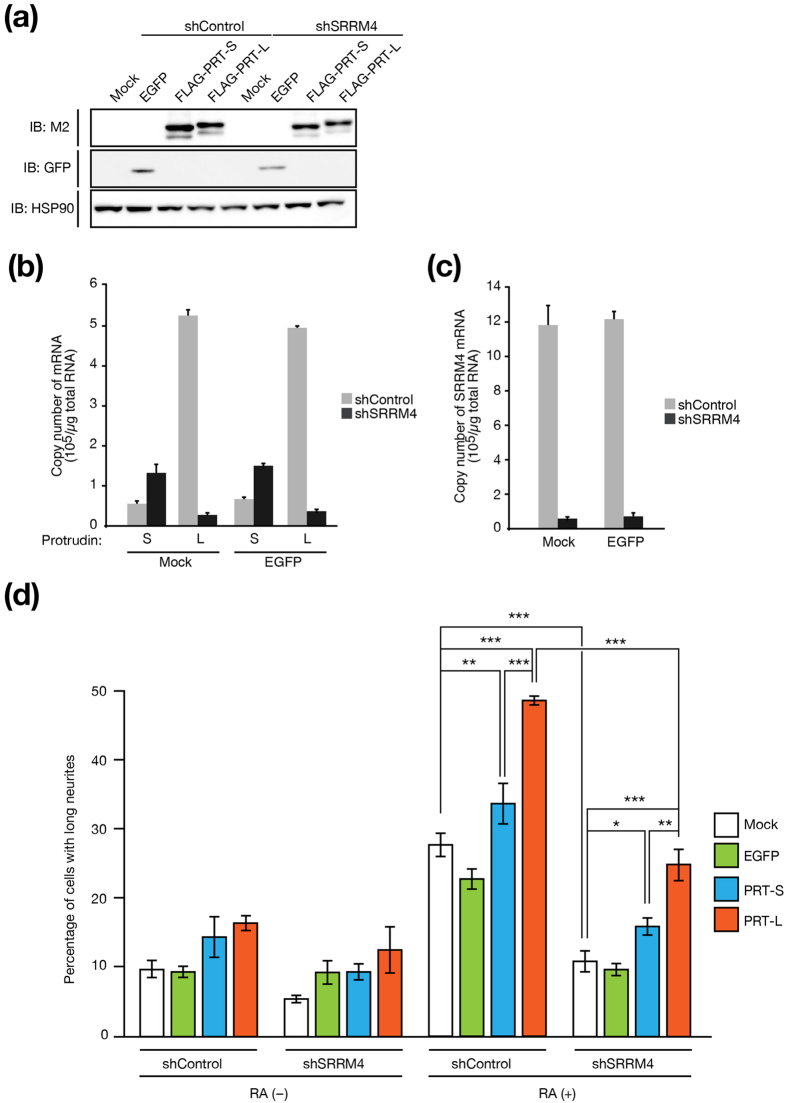
Protrudin-L restores neurite formation in SRRM4-depleted cells. (**a**) Neuro2A cells infected with retroviruses encoding control or SRRM4 shRNAs as well as with those encoding enhanced GFP (EGFP), FLAG–protrudin-S (PRT-S), or FLAG–protrudin-L (PRT-L) were subjected to immunoblot analysis with antibodies to FLAG (M2), to GFP, and to HSP90 (loading control). (**b**, **c**) Quantitative RT-PCR analysis of the absolute amounts of protrudin-S and protrudin-L mRNAs (**b**) as well as of SRRM4 mRNA (**c**) in Neuro2A cells infected with retroviruses encoding control or SRRM4 shRNAs as well as with a retrovirus encoding EGFP or with the corresponding empty virus (Mock). Data are means ± s.e.m. from three independent experiments. (**d**) Quantification of the percentage of Neuro2A cells infected with retroviruses as in **a** that extended long neurites (greater than three cell body lengths) during culture in the absence or presence of RA for 48 h. Data are means ± s.e.m. for three independent experiments. A total of 11,993 cells was examined. *P < 0.05, **P < 0.01, ***P < 0.001 (one-way ANOVA followed by the Tukey-Kramer test).

Neuro2A cells extend neurites in response to treatment with retinoic acid (RA) for 48 h, and this extension is inhibited by depletion of SRRM4 (ref. 21). We therefore next examined whether such impairment of neurite outgrowth by SRRM4 depletion might be rescued by forced expression of protrudin-L (Fig. 4b,c). Although the expression levels of FLAG–protrudin-S and FLAG–protrudin-L in Neuro2A cells were similar (Fig. 4a), such overexpression of protrudin-L stimulated neuritogenesis to a much greater extent than did that of protrudin-S in control or SRRM4-depleted cells exposed to RA (Fig. 4d). Whereas ~50% of RA-treated Neuro2A cells expressing the control shRNA and FLAG–protrudin-L extended long neurites, only ~25% of the corresponding SRRM4-depleted cells did so, suggesting that SRRM4 might also control neurite extension by targeting other factors.

**Figure 5:**
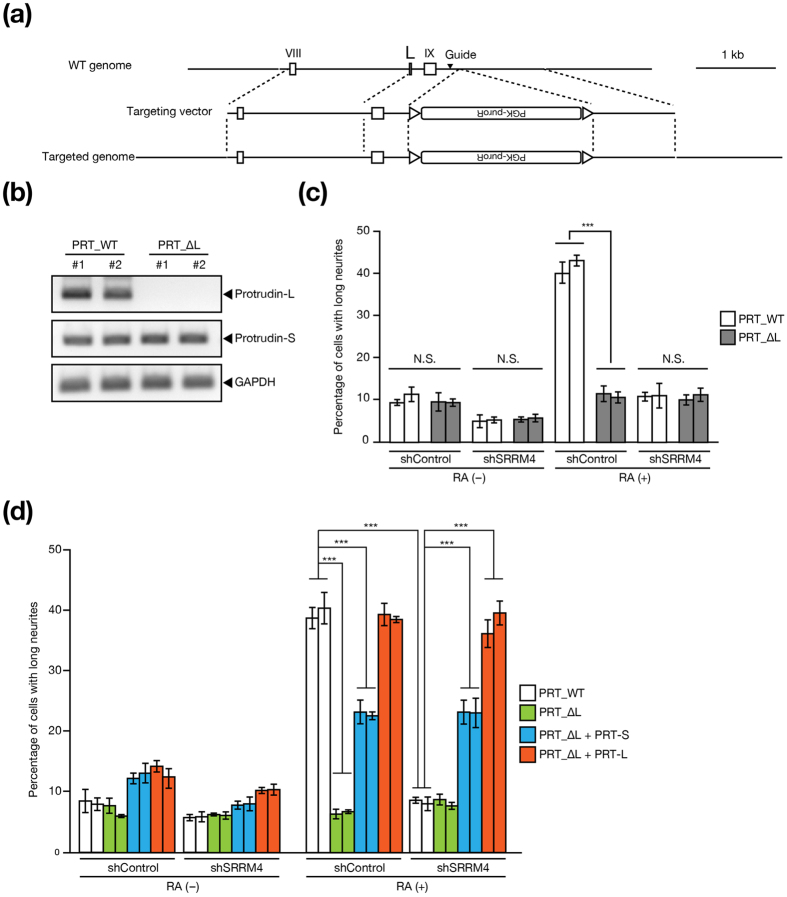
Exon L of the protrudin gene contributes to RA-induced neurite outgrowth in Neuro2A cells. (**a**) Structure of the mouse protrudin gene locus as well as of the targeting vector for deletion of exon L and the targeted allele after homologous recombination mediated by the CRISPR-Cas9 system. The filled arrowhead indicates the position of the guide RNA for Cas9. (**b**) RT-PCR analysis of protrudin transcripts in WT (PRT_WT) and exon L–deleted (PRT_∆L) Neuro2A cells. (**c**) Quantification of the percentage of PRT_WT or PRT_∆L Neuro2A cells that extended long neurites (greater than three cell body lengths) during culture in the absence or presence of RA for 48 h. The cells were also infected with retroviruses encoding control or SRRM4 shRNAs as indicated. (**d**) Quantification of neurite formation for Neuro2A cells as in **c** with the addition that PRT_∆L cells infected with retroviruses encoding protrudin-S (PRT-S) or protrudin-L (PRT-L) as indicated were also examined. Results are shown for two independent lines of WT and exon-deleted cells in (**b)** to (**d)**. Data in (**c** and **d)** are means ± s.e.m. for three independent experiments. ***P < 0.001 (one-way ANOVA followed by the Tukey-Kramer test).

To evaluate further the role of exon L of the protrudin gene in RA-induced neuritogenesis in Neuro2A cells, we deleted this exon (PRT_∆L) by homologous recombination with the CRISPR-Cas9 system (Fig. 5a,b). As a control, we also generated Neuro2A cells that retain exon L (PRT_WT) with the use of this system. Deletion of exon L greatly attenuated the formation of neurites in response to RA treatment in control Neuro2A cells, whereas it had little effect in SRRM4-depleted cells (Fig. 5c). We also examined the effect of overexpression of protrudin-S or protrudin-L in Neuro2A cells in which exon L was disrupted. The extent of RA-induced neuritogenesis was almost fully restored in control or SRRM4-depleted Neuro2A (PRT_ΔL) cells by overexpression of protrudin-L whereas overexpression of protrudin-S had a similar but smaller effect (Fig. 5d). Deletion of a microexon of *Apbb1*, a target gene of SRRM4 that also plays a role in neurite outgrowth^29^,^30^, had no effect on RA-induced neuritogenesis (Supplementary Fig. 1). These results suggested that exon L of the protrudin gene is essential for RA-induced neurite outgrowth in Neuro2A cells, and that protrudin-L is the main downstream mediator of SRRM4 action in the regulation of neurite outgrowth.

### Exon L of the protrudin gene contributes to polarity formation during neurogenesis in ESCs

**Figure 6:**
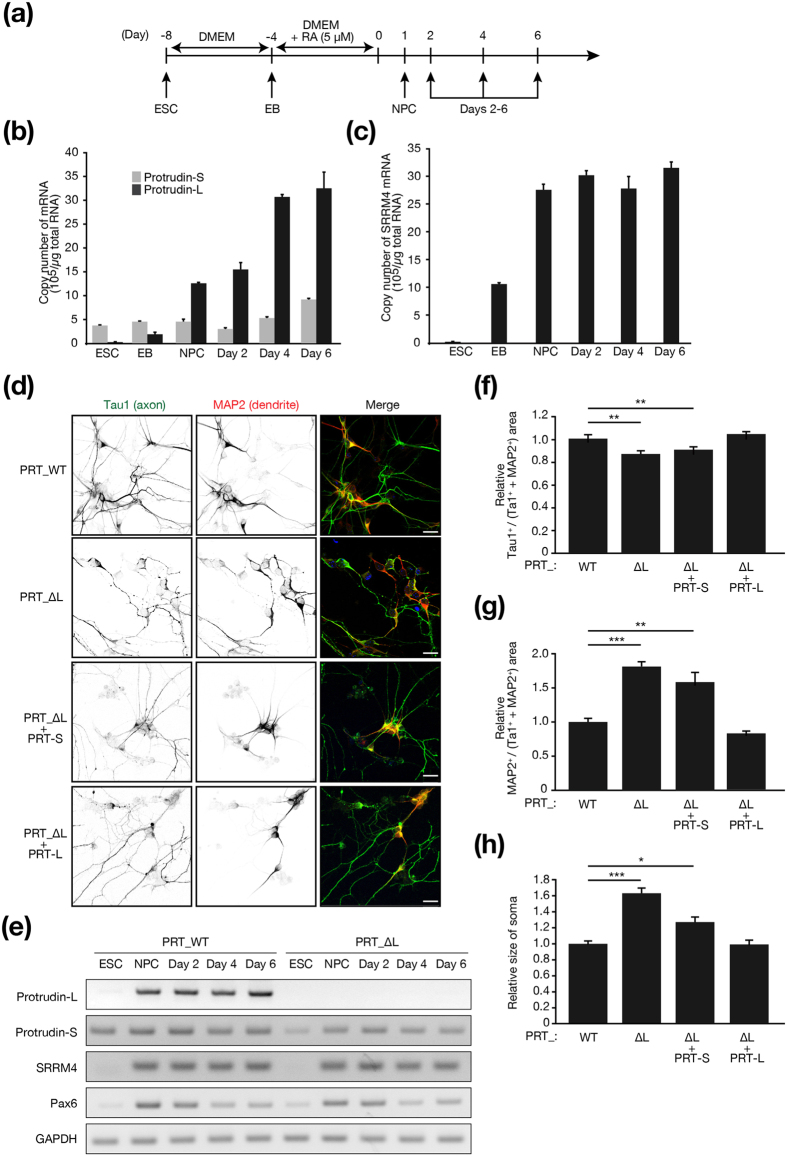
Protrudin-L contributes to polarity formation during neurogenesis in ESCs. (**a**) Experimental protocol for the induction of neuronal differentiation in ESCs. ESCs were cultured in Dulbecco’s modified Eagle’s medium (DMEM) without feeder cells for 4 days and then treated with 5 μM RA for the next 4 days. Embryoid bodies (EBs) formed from the ESCs were dissociated and transferred to dishes coated with ornithine and laminin. (**b**,**c**) Quantitative RT-PCR analysis of the absolute amounts of protrudin-S and protrudin-L mRNAs (**b**) as well as of SRRM4 mRNA (**c**) during ESC differentiation. Data are means ± s.e.m. of triplicates from a representative experiment. (**d**) Neurons differentiated either from ESCs lacking exon L of the protrudin gene (PRT_ΔL) and expressing (or not) exogenous protrudin-S (PRT-S) or protrudin-L (PRT-L) or from corresponding WT (PRT_WT) cells at day 2 after termination of RA treatment were fixed and processed for immunofluorescence analysis with antibodies to Tau1 (green) and to MAP2 (red). Merged images in which nuclei are stained with Hoechst 33258 (blue) are also shown. Scale bars, 20 μm. (**e**) RT-PCR analysis of the indicated mRNAs during differentiation of PRT_WT and PRT_ΔL ESCs. (**f**–**h**) Relative Tau1-positive (**f**) or MAP2-positive (**g**) area as well as relative size of the soma (**h**) for neurons derived from ESCs as determined with ImageJ software from images similar to those in (**d**). Data are means ± s.e.m. for five randomized areas (**f**,**g**) or for 55 (PRT_WT), 54 (PRT_∆L), 44 (PRT_∆L + PRT-S), or 46 (PRT_∆L + PRT-L) neurons (**h**). *P < 0.05, **P < 0.01, ***P < 0.001 (Student’s *t* test).

Neurogenesis is recapitulated by the induction of neuronal differentiation in ESCs (Fig. 6a). We measured the abundance of protrudin-S, protrudin-L, and SRRM4 mRNAs during such differentiation of mouse ESCs (Fig. 6b,c). Whereas the amount of protrudin-S mRNA remained relatively constant, that of protrudin-L and SRRM4 mRNAs increased with differentiation progression, with the increase in SRRM4 mRNA preceding that for protrudin-L mRNA.

We next generated ESCs that lack exon L of the protrudin gene (PRT_∆L) with the use of the CRISPR-Cas9 system and confirmed the disruption of exon L in neurons derived from PRT_∆L ESCs by RT-PCR analysis (Fig. 6e). We also generated ESCs that retain exon L (PRT_WT) with the CRISPR-Cas9 system as a control. The abundance of protrudin-S, SRRM4, and Pax6 mRNAs did not differ between neurons derived from PRT_WT or PRT_∆L ESCs. Two days after the termination of RA treatment to induce neuronal differentiation, we subjected the neurons derived from PRT_WT or PRT_∆L ESCs to immunofluorescence analysis of Tau1 and microtubule-associated protein 2 (MAP2), specific markers for axons and dendrites, respectively (Fig. 6d). The ratio of the Tau1-positive area to the sum of the Tau1- or MAP2-positive areas was significantly reduced for neurons derived from PRT_∆L ESCs compared with those derived from PRT_WT ESCs (Fig. 6f), whereas the corresponding ratio for the MAP2-positive area was increased (Fig. 6g). Furthermore, neurons derived from PRT_∆L ESCs had larger somas than those derived from PRT_WT ESCs (Fig. 6h). These phenotypes of neurons derived from PRT_∆L ESCs were almost fully rescued by overexpression of protrudin-L but not by that of protrudin-S (Fig. 6d,f–h). These results indicated that the lack of exon L of the protrudin gene impairs the development of neuronal polarity associated with polarized intracellular transport during early neurogenesis. The SRRM4-mediated production of protrudin-L thus plays an important role in polarity formation during neurogenesis.

## Discussion

We have here shown that SRRM4, a splicing regulator specifically expressed in neurons, promotes the neuron-specific alternative splicing of protrudin pre-mRNA to produce protrudin-L, which in turn contributes to neurite outgrowth during neurogenesis.

Ablation of SRRM4 in the developing mouse cortex was previously shown to inhibit neuronal differentiation and to result in the accumulation of Pax6-positive progenitor cells in the ventricular zone and a reduction in the number of differentiated cells in the cortical plate^23^. SRRM4-deficient mice also manifest fewer late-born, upper-layer neurons and more early-born, lower-layer neurons compared with WT mice, suggestive either of early depletion of the NPC pool or altered neuronal subtype specification^17^,^31^. These observations thus implicated SRRM4 in neurogenesis *in vivo*.

We have now shown that SRRM4 may contribute to recognition of a UGC motif located in the polypyrimidine tract between the branch point and 3′ splice site of the intron upstream of exon L of protrudin pre-mRNA and thereby promote inclusion of exon L in the mature transcript in neural cells. Docking of U2AF65 and U2AF35 to the polypyrimidine tract and 3′ splice site, respectively, are essential steps of the splicing reaction. Whereas a typical acceptor sequence may recruit splicing factors in a manner independent of SRRM4, the atypical acceptor sequence flanking exon L of protrudin pre-mRNA may repress the inclusion of exon L in nonneuronal cells. Given that SRRM4 is absent in nonneuronal cells, the atypical acceptor sequence is likely unable to mediate the splicing reaction at exon L, resulting in the generation of protrudin-S. Protrudin-L promotes neurite outgrowth, and its overexpression rescued, at least in part, the impairment of RA-induced neurite formation in SRRM4-depleted Neuro2A cells. Furthermore, neurons derived from ESCs lacking exon L of the protrudin gene manifested a reduced axon/dendrite ratio, providing further support for the notion that protrudin-L plays an important role in both neurite outgrowth and polarity formation. Neuritogenesis was previously shown to be impaired in neurons lacking exons 7 and 8 of the SRRM4 gene, and forced expression of a form of UNC13B that includes the region encoded by a microexon targeted by SRRM4 in these neurons restored the length of neurites to that observed in WT neurons^17^. UNC13B participates in the priming stem of synaptic vesicle exocytosis and early neurite extension^32^,^33^,^34^. Neuritogenesis requires a supply of lipids, and vesicles containing lipids are transported to and fuse with the membrane of the growth cone. SRRM4 thus appears to coordinately regulate protrudin-L and UNC13B, which contribute to the transport and fusion of such vesicles, respectively.

Whereas SRRM4 regulates the inclusion of neural-specific microexons of many genes, it also contributes to transcriptional events through RE-1 silencing transcription factor (REST). SRRM4 promotes the inclusion of the neural exon of the REST gene and thereby generates the isoform REST-4, which lacks four zinc fingers and a COOH-terminal repressor domain that are required for full DNA binding and repressive activity^35^,^36^. Chromatin immunoprecipitation and other analyses have shown that mouse REST occupies multiple RE-1 sites within the SRRM4 gene and that this gene is a transcriptional target of REST^23^,^37^. It is thus possible that REST or other transcription factors that are downstream targets of SRRM4 play a role in transcription of the protrudin gene. SRRM4 might therefore contribute not only to microexon selection but also to transcriptional elongation or efficiency at the protrudin gene. We speculate that overexpression of SRRM4 might increase the total abundance of protrudin pre-mRNA, which might then be efficiently converted to protrudin-L mRNA without a change in the abundance of protrudin-S mRNA.

Impairment of neuritogenesis is thought to increase the risk of mental disorders^38^. For instance, the interaction of DISC1 with FEZ1 is essential for neurite outgrowth during the differentiation of PC12 cells, with expression of a truncated form of DISC1 inhibiting neurite extension^39^. Given that mutation of *DISC1* is implicated in schizophrenia, the pathogenesis of this condition may be related to impaired neuritogenesis. Furthermore, the inclusion of many microexons targeted by SRRM4 in mature transcripts is dysregulated in individuals with autism spectrum disorder (ASD), a neurodevelopmental disorder characterized by impaired social interaction, social communication, and imaginative thought^24^. Impairment of SRRM4 function might thus contribute to the etiology of ASD. The expression level of SRRM4 has indeed been found to be reduced in some ASD patients^24^. In addition, genes that have been associated with ASD also include *PRKD2* and *PPCS2*, both of which contribute to neuritogenesis^40^.

The protrudin gene has been found to be mutated in a subset of individuals with the autosomal dominant form of hereditary spastic paraplegia (AD-HSP), which is characterized by selective degeneration of axons^41^,^42^. Protrudin interacts with Rab11, KIF5, and VAP, all of which are associated with vesicular trafficking^25^,^26^,^27^,^28^. The amino acids encoded by exon L of the protrudin gene are located adjacent to an FFAT motif that is required for the binding to VAP, and SRRM4-mediated inclusion of exon L in protrudin mRNA indeed supports the binding of protrudin to VAP and consequent promotion of neurite outgrowth^28^. Although SRRM4 regulates alternative splicing of many genes, we found that its regulation of alternative splicing of exon L of the protrudin gene is essential for SRRM4-dependent neurite extension. Impaired inclusion of exon L of the protrudin gene might therefore be central to disorders attributable to loss of function of SRRM4. Comparison of the phenotypes of SRRM4-deficient mice with those of mice lacking exon L of the protrudin gene should provide further insight into the functional relation between SRRM4 and protrudin.

## Methods

### Construction of expression plasmids

Mouse cDNA encoding SRRM4 (GenBank accession number NM_026886.3) was amplified from Neuro2A cell cDNA by PCR with PrimeSTAR HS DNA polymerase (TaKaRa, Shiga, Japan) and specific primers, and it was subcloned into the pMX-puro vector. Construction of vectors encoding mouse protrudin-S or protrudin-L was described previously^28^.

### Construction of protrudin splicing minigenes

A genomic region spanning exons VIII to IX of the mouse protrudin gene was amplified by PCR and cloned into a pMX-puro vector encoding mVenus (GFP). Mutation of the 3′ splice site flanking exon L was achieved by PCR.

### Cell culture, transfection, and infection and quantification of neurite formation

Neuro2A cells were cultured under a humidified atmosphere of 5% CO_2_ at 37 °C in DMEM (Invitrogen, Carlsbad, CA) supplemented with 10% fetal bovine serum (Invitrogen). NIH 3T3 cells were similarly cultured in DMEM supplemented with 10% calf serum (Invitrogen). For retrovirus production, Plat E cells were transiently transfected with pMX-puro–based vectors with the use of the FuGene HD reagent (Promega, Fitchburg, WI) and then cultured for 48 h. The retroviruses released into the culture medium were then used to infect Neuro2A or NIH 3T3 cells. Differentiation of Neuro2A cells was induced by culture in DMEM supplemented with 2% fetal bovine serum and 20 μM all-*trans* retinoic acid for 48 h. The proportion of Neuro2A cells that extended long neurites (those longer than three cell body lengths) was quantified.

### Deletion of microexons of the protrudin gene and *Apbb1* with the CRISPR-Cas9 system

Complementary oligonucleotides containing protrudin single guide RNA (sgRNA) target sequences were annealed and cloned into the BbsI site of pX330 (Addgene, Cambridge, MA). The target sequences of the protrudin primers (sense and antisense, respectively) were 5′-CACCGTAGCTCAACTAGTGTCCTT-3′ and 5′-AAACAAGGACACTAGTTGAGCTAC-3′ for mouse protrudin-L #1 and 5′-CACCGTGTAGCTCAACTAGTGTCCT-3′ and 5′-AAACAGGACACTAGTTGAGCTACAC-3′ for mouse protrudin-L #2. The target sequences of the *Apbb1* primers (sense and antisense, respectively) were 5′-CACCGAGTCCTTCTCGGGATGCTCG-3′ and 5′-AAACCGAGCATCCCGAGAAGGACTC-3′ for mouse *Apbb1* #1 and 5′-CACCGAGCCACGAGCATCCCGAGA-3′ and 5′-AAACTCTCGGGATGCTCGTGGCTC-3′ for mouse *Apbb1* #2. For generation of the targeting vector for homologous recombination, a puromycin resistance gene controlled by the mouse phosphoglycerate kinase gene promoter (PGK-*puro*^R^) and flanked by 5′ and 3′ regions of homology containing 2 kbp upstream and 1 kbp downstream of the cleavage site in the intron downstream of exon IX of the protrudin gene was incorporated into pBSII-SK + (Addgene). Similarly, PGK-*puro*^R^ flanked by 5′ and 3′ regions of homology containing 1 kbp upstream and 1 kbp downstream of the cleavage site in the intron of *Apbb1* was separately incorporated into pBSII-SK + . The sgRNA and targeting vectors were introduced into Neuro2A cells by transfection with the use of the FuGene HD reagent (Promega), and the cells were subjected to selection in medium containing puromycin (3 μg/ml). They were then diluted and transferred to 96-well plates to obtain single cell–derived clones in which both alleles had undergone homologous recombination.

### Qualitative and quantitative RT-PCR analysis

Total RNA was isolated from cells with the use of Isogen (Nippon Gene, Tokyo, Japan), portions of the RNA were subjected to RT with the use of a Quantitect Kit (Qiagen, Venlo, the Netherlands), and the resulting cDNA was subjected either to PCR with Taq polymerase (TaKaRa) and specific primers followed by agarose gel electrophoresis or to real-time PCR with the use of a StepOnePlus real-time PCR system (Applied Biosystems, Foster City, CA), SYBR Premix Ex *Taq* (TaKaRa), and specific primers. PCR primer sequences (sense and antisense, respectively) were as follows: 5′-AAAGATGCAATTGAGGAGGA-3′ and 5′-TCTTCTTCAGCACGGAGAACG-3′ for mouse protrudin-S, 5′-AGACCCACCTGGTGGTGCTG-3′ and 5′-ACACACACACAGTCTCTCTC-3′ for mouse protrudin-L, 5′-CAGTATCACAGCCCGCAAG-3′ and 5′-CCTGATCTGGGAGCAAGAGA-3′ for mouse SRRM4, 5′-CATGGCCTTCCGTGTTCCTA-3′ and 5′-GCGGCACGTCAGATCCA-3′ for mouse GAPDH, 5′-TACCAGTGTCTACCAGCCAAT-3′ and 5′-TGCACGAGTATGAGGAGGTCT-3′ for mouse Pax6, 5′-CAAAGTCATGCCTACAAAGTCTCC-3′ and 5′-AGACACTACAGGCGTGGCAAGA-3′ for Mef2a, 5′-GTCACCAGAACCATCCAGAATAACTT-3′ and 5′-GTCTGGTTCTTGAACGCTATGCT-3′ for Synj1, 5′-CTCATGCTGCAGTTCTCGAGTTGTCCTGC-3′ and 5′-CTTCATGTGGTCGGGGTAGC-3′ for protrudin pre-mRNA (long), 5′-CAGAGCTCATCAGGCACAGCTG-3′ and 5′-CACCAAGTGTGGCACAGCTAACTC-3′ for protrudin pre-mRNA (short), and 5′-ACCCATGTAAGAGGAGGTGGGATTAAG-3′ and 5′-CTGTGTTACGATCTCAGGGACTCTGAG-3′ for Mef2a pre-mRNA, and 5′-AACCATGGACAGTGTTCGGTCT-3′ and 5′-TATAGTGCCCTTTGGCCCAGTT-3′ for Tuj1 pre-mRNA.

### Antibodies

Antibodies to GFP (for detection of EGFP) were obtained from Frontier Science (Hokkaido, Japan); those to HSP90 were from BD Biosciences (San Jose, CA); those to FLAG (M2) and to U2AF65 were from Sigma (St. Louis, MO); those to Tau1 were from Millipore (Billerica, MA); those to U2AF35 were from Bethyl Laboratories (Montgomery, AL); those to MAP2 were from Abcam (Cambridge, MA); and those to c-Jun were from Cell Signaling Technology (Danvers, MA). Horseradish peroxidase–conjugated goat antibodies to mouse or rabbit immunoglobulin G were from Promega.

### RNA interference

Construction of shRNA vectors was performed as described previously^43^. In brief, the pMX-puro II vector was constructed by deletion of the U3 portion of the 3′ long terminal repeat of pMX-puro. The mouse U6 gene promoter, followed by DNA corresponding to an shRNA sequence, was then subcloned into the NotI and XhoI sites of pMX-puro II, yielding pMX-puro II-U6/siRNA. The DNA for each shRNA encoded a 21-nucleotide hairpin sequence specific to the mRNA target, with a loop sequence (-TTCAAGAGA-) separating the two complementary domains, and it also contained a tract of five T nucleotides to terminate transcription. The resulting vectors were used to transfect Plat E cells and thereby to generate recombinant retroviruses. The sequence targeted for mouse SRRM4 was 5′-CTCACAGTATATCTCCTAAAC-3′. Silencer Select siRNAs designed for mouse U2AF35 (U2AF1, 5′-UGUGCAACCGAGAACAUCGT-3′) or U2AF65 (U2AF2, 5′-UCAUCAUUUAGGUAAUUCGGC-3′) or a negative control duplex (Thermo Fisher Scientific, San Jose, CA) were introduced into Neuro2A cells by transfection with the use of Lipofectamine RNAiMax (Invitrogen).

### RNA immunoprecipitation

RNA immunoprecipitation was performed as described previously^44^. In brief, Neuro2A cells (1 × 10^7^) expressing both FLAG-SRRM4 and either WT or tac-1 versions of the protrudin minigene were harvested by exposure to trypsin, resuspended in a mixture of 2 ml of phosphate-buffered saline, 2 ml of nuclear isolation buffer (1.28 M sucrose, 40 mM Tris-HCl (pH 7.5), 20 mM MgCl_2_, 4% Triton X-100), and 6 ml of water, and then incubated on ice for 20 min (with mixing every 5 min). Nuclei were isolated by centrifugation at 2,500 × *g* for 15 min at 4 °C, resuspended in 1 ml of RIP buffer (150 mM KCl, 25 mM Tris-HCl (pH 7.5), 5 mM EDTA, 0.5 mM dithiothreitol, 0.5% Nonidet P-40, leupeptin (9 μg/ml), aprotinin (3 μg/ml), 1 mM phenylmethylsulfonyl fluoride, and RNase inhibitor (100 U/ml; TOYOBO, Tokyo, Japan)), and mechanically sheared with the use of a Dounce homogenizer (15 strokes). Nuclear membrane and debris were removed by centrifugation at 15,300 × *g* for 10 min at 4 °C, and the resulting supernatant was incubated for 1 h at 4 °C (with gentle rotation) with protein G beads conjugated with M2 antibodies to FLAG (Sigma). The beads were isolated by centrifugation at 500 × *g* for 1 min, resuspended in 500 μl of RIP buffer, and washed an additional two times with RIP buffer and once with phosphate-buffered saline. They were finally resuspended in 1 ml of Isogen (Nippon Gene) for isolation of bound RNA, which was then subjected to RT-PCR analysis of protrudin and Mef2a mRNAs. Bead-bound proteins were also detected by immunoblot analysis.

### ESC differentiation

Differentiation of mouse ESCs was performed essentially as described. In brief, ESCs were deprived of feeder cells for three or four passages, after which 4 × 10^6^ of the cells were allowed to form EBs during culture in nonadherent bacterial dishes for 8 days, with all-*trans* retinoic acid (5 μM) being added from days 4 to 8. The EBs were then dissociated and transferred to dishes coated with ornithine and laminin.

### Immunofluorescence analysis and analysis of cell areas

Cells were fixed for 10 min at 37 °C with 4% paraformaldehyde in phosphate-buffered saline, incubated consecutively with primary antibodies and Alexa Fluor 488– or Alexa Fluor 546–labeled secondary antibodies (Thermo Fisher Scientific) in phosphate-buffered saline containing 0.5% Triton X-100, and covered with a drop of GEL/MOUNT (Biomeda, Hayward, CA) before examination with a confocal fluorescence microscope (LSM700; Carl Zeiss, Oberkochen, Germany). Cell areas were analyzed with the use of ImageJ software.

### Statistical analysis

Quantitative data are presented as means ± s.e.m. and were analyzed with Student’s *t* test or by one-way analysis of variance (ANOVA) followed by the Tukey-Kramer multiple-comparison test. A *P* value of < 0.05 was considered statistically significant.

## Additional Information

**How to cite this article**: Ohnishi, T. *et al*. SRRM4-dependent neuron-specific alternative splicing of protrudin transcripts regulates neurite outgrowth. *Sci. Rep.* **7**, 41130; doi: 10.1038/srep41130 (2017).

**Publisher's note:** Springer Nature remains neutral with regard to jurisdictional claims in published maps and institutional affiliations.

## Supplementary Material

Supplementary Information
