# Supplementary material for: SRRM4-dependent neuron-specific alternative splicing of protrudin transcripts regulates neurite outgrowth
Source: Sci Rep. 2017 Jan 20;7:41130. doi: 10.1038/srep41130 (PMC5247714; doi:10.1038/srep41130)
Supplement: Supplementary Information [file srep41130-s1.pdf]

Supplementary Information

SRRM4-dependent neuron-specific alternative splicing of protrudin transcripts regulates neurite outgrowth

Takafumi Ohnishi, Michiko Shirane*, and Keiichi I. Nakayama

Department of Molecular and Cellular Biology, Medical Institute of Bioregulation, Kyushu University, 3-1-1 Maidashi, Higashi-ku, Fukuoka, Fukuoka 812-8582, Japan.

*Corresponding author. Department of Molecular and Cellular Biology, Medical Institute of Bioregulation, Kyushu University, 3-1-1 Maidashi, Higashi-ku, Fukuoka, Fukuoka 812-8582, Japan. Tel.: +81-92-642-6816. Fax: +81-92-642-6819. E-mail: smichi@bioreg.kyushu-u.ac.jp

Supplementary Figure Legends

Supplementary Figure 1–3

Supplementary Figure Legends

Supplementary Figure 1 | Microexon of the Appb1 gene does not contribute to RA-induced neurite outgrowth in Neuro2A cells.
(**a**) Structure of the mouse Apbb1 gene locus as well as the targeting vector for deletion of the microexon and the targeted allele resulting from homologous recombination mediated by the CRISPR-Cas9 system. The filled arrowhead indicates the position of the guide RNA for Cas9. (**b**) Direct sequencing analysis of *Apbb1* genomic DNA from WT or microexon-deleted (Δexon) Neuro2A cells. The boxed region in the upper panel corresponds to the deleted microexon in the lower panel. (**c**) Quantification of the percentage of WT or Δexon Neuro2A cells that extended long neurites (greater than three cell body lengths) during culture in the absence or presence of RA for 48 h. The cells were also infected with retroviruses encoding control or SRRM4 shRNAs as indicated. Data in **c** are means ± s.e.m. for three independent experiments. ***$P < 0.001$ (one-way ANOVA followed by the Tukey–Kramer test). Results are shown for two independent lines of WT and exon-deleted cells in **c**.

Supplementary Figure 2 | Full scans of key immunoblots in Figure 1 and 2.

Supplementary Figure 3 | Full scans of key immunoblots in Figure 3–6.

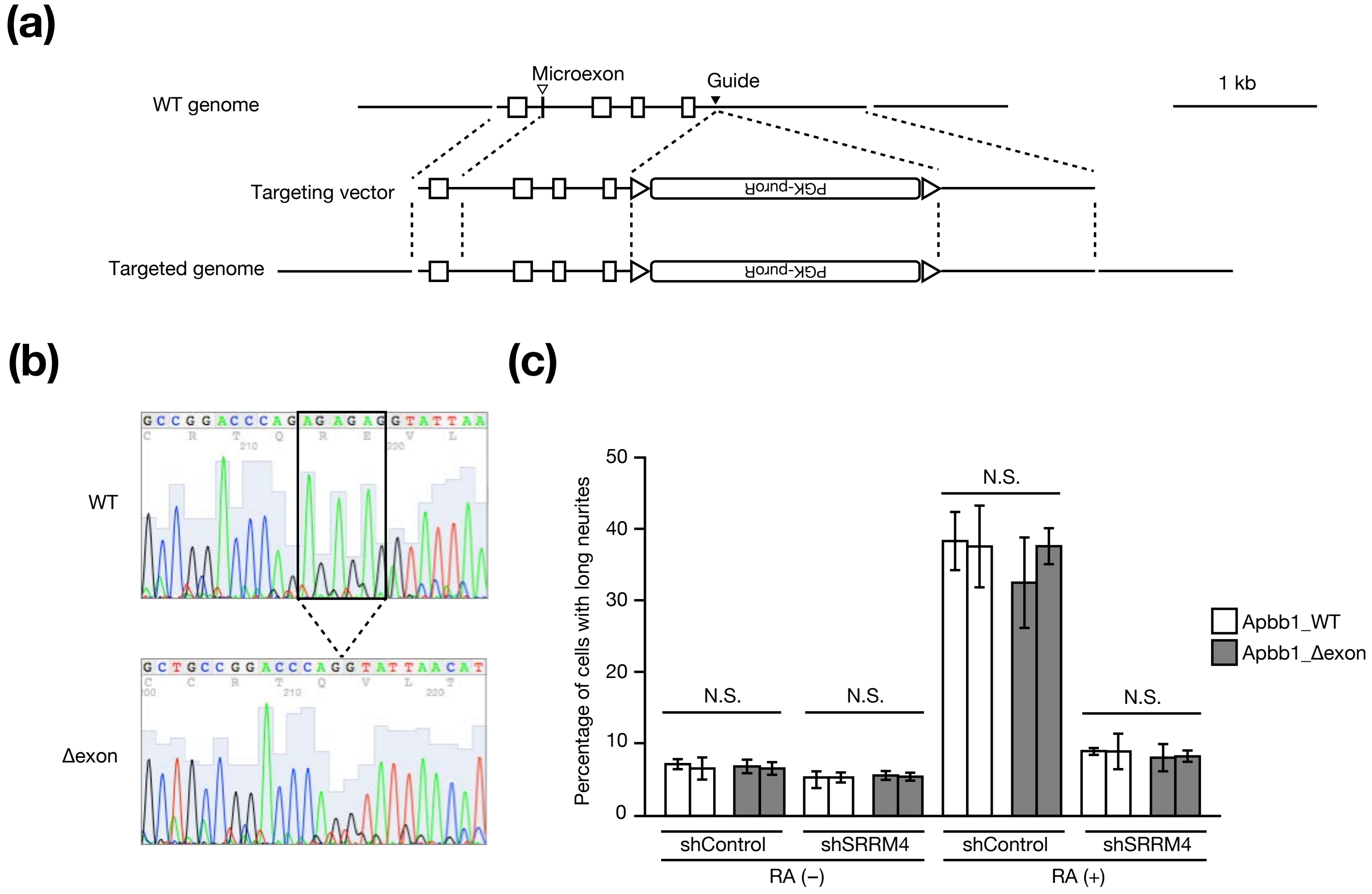

Supplementary Figure 1

Fig. 1c

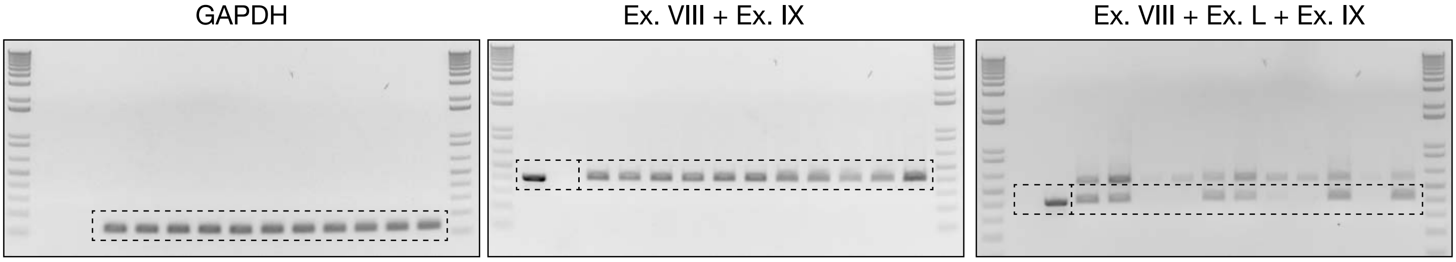

Fig. 2c

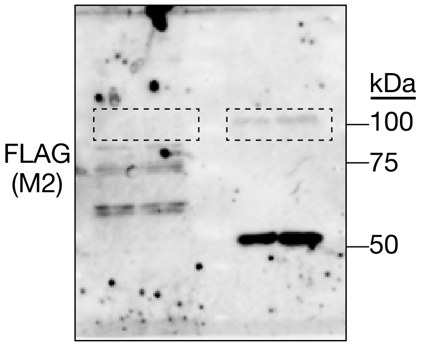

Fig. 2c

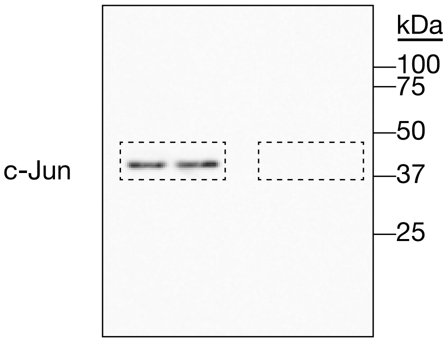

Fig. 2c

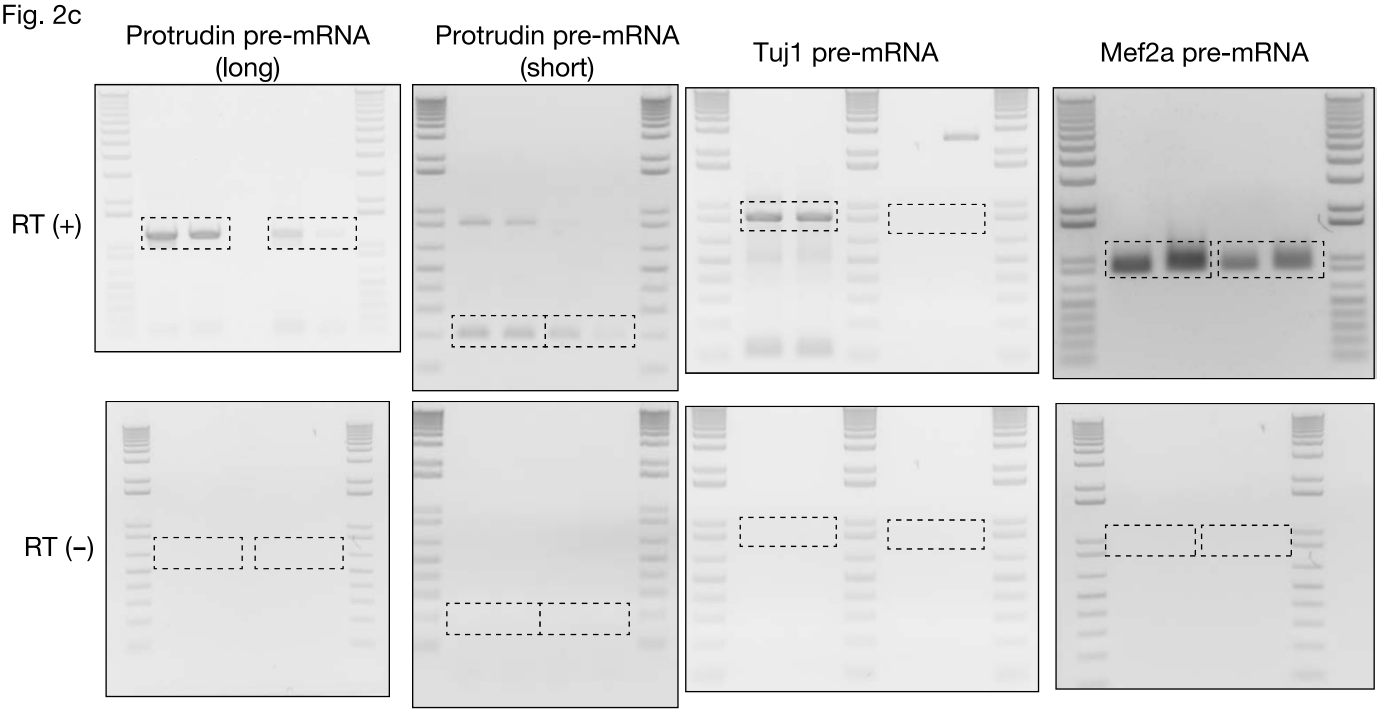

Fig. 2e

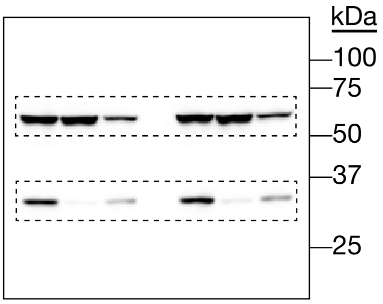

IB:U2AF35, U2AF65

Fig. 2e

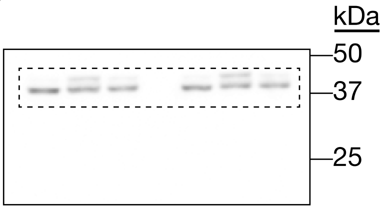

IB:c-Jun

Fig. 2f

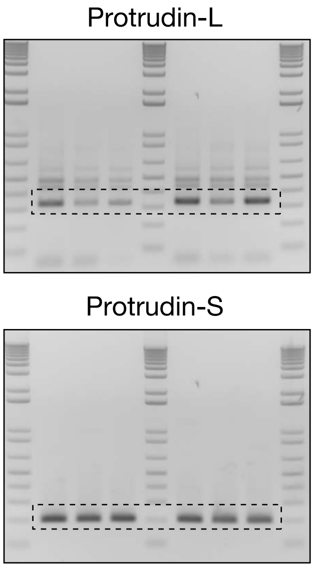

Supplementary Figure 2

Fig. 3c

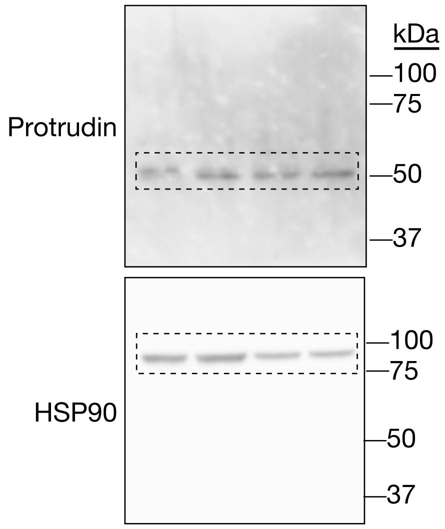

Fig. 3d

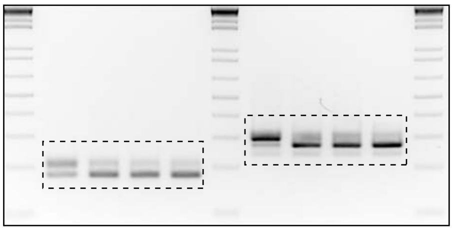

Fig. 3g

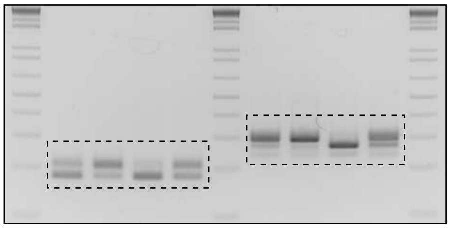

Fig. 4a

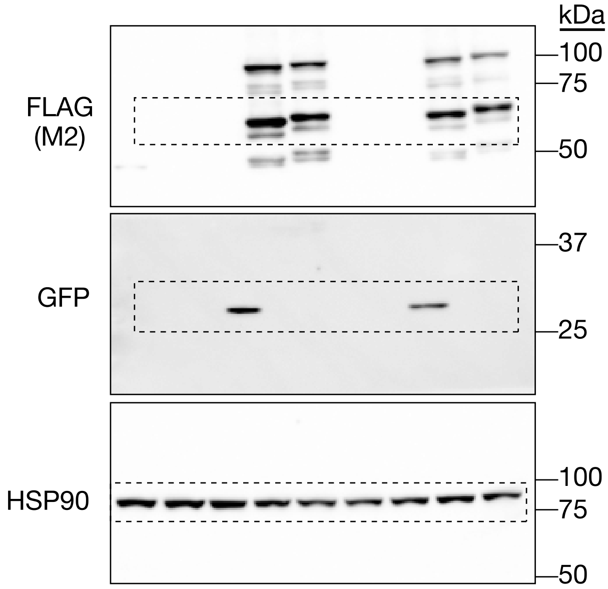

Fig. 5b

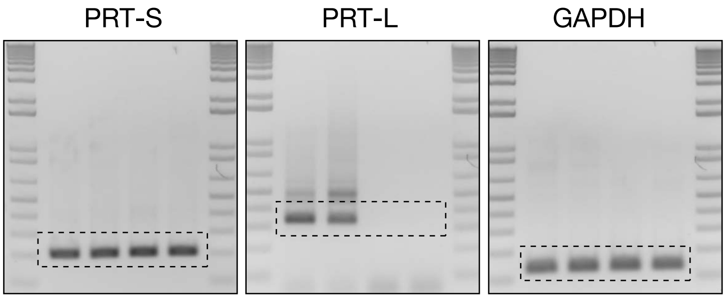

Fig. 6e

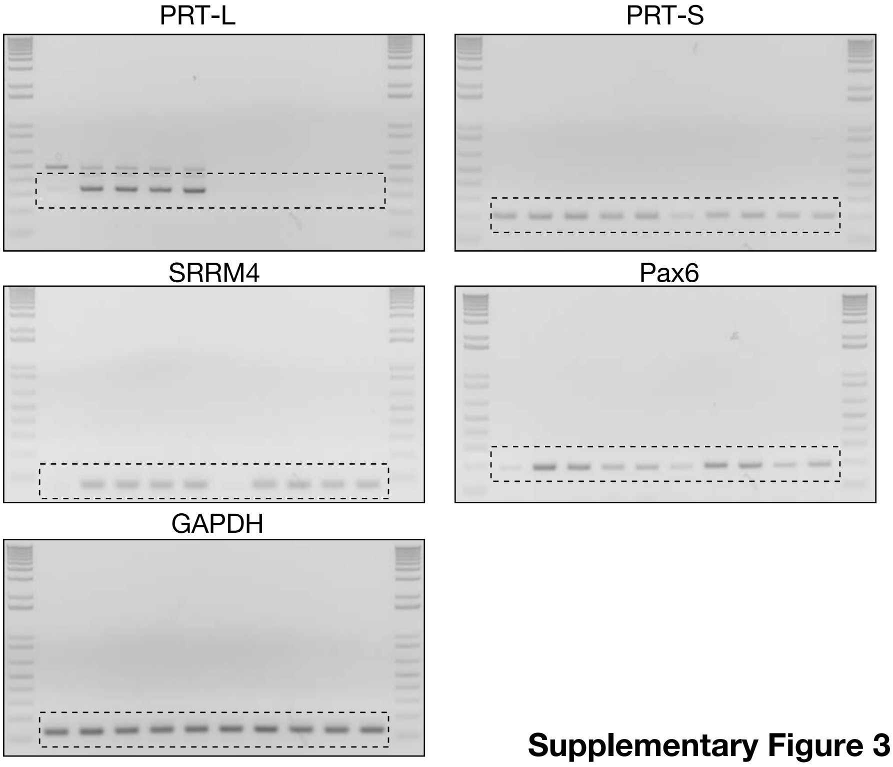

Supplementary Figure 3
